# Modification of graphene oxide film properties using KrF laser irradiation

**DOI:** 10.1039/c8ra00097b

**Published:** 2018-04-03

**Authors:** Somayeh Mortazavi, Mahmoud Mollabashi, Rasoul Barri, Kevin Jones, John Q. Xiao, Robert L. Opila, S. Ismat Shah

**Affiliations:** School of Physics, Iran University of Science and Technology Tehran 16844 Iran mollabashi@iust.ac.ir +98 7302 1452 +98 7322 5858; Department of Physics & Astronomy, University of Delaware Newark DE 19716 USA ismat@udel.edu; Department of Materials Sciences and Engineering, University of Delaware Newark DE 19716 USA; Department of Electrical and Computer Engineering, University of Delaware Newark DE 19716 USA

## Abstract

Modification of various properties of graphene oxide (GO) films on SiO_2_/Si substrate under KrF laser radiation was extensively studied. X-ray diffraction, X-ray photoelectron spectroscopy, Raman spectroscopy and the electrical resistance measurements were employed to correlate the effects of laser irradiation on structural, chemical and electrical properties of GO films under different laser fluences. Raman spectroscopy shows reduced graphene oxide patterns with increased *I*_2D_/*I*_G_ ratios in irradiated samples. X-ray photoelectron spectroscopy shows a high ratio of carbon to oxygen atoms in the reduced graphene oxide (rGO) films compared to the pristine GO films. X-ray diffraction patterns display a significant drop in the diffraction peak intensity after laser irradiation. Finally, the electrical resistance of irradiated GO films reduced by about four orders of magnitudes compared to the unirradiated GO films. Simultaneously, reduction and patterning of GO films display promising fabrication technique that can be useful for many graphene-based devices.

## Abstract 2

Modification of various properties of graphene oxide (GO) films on SiO_2_/Si substrate under KrF laser radiation was extensively studied.

## Graphical abstract

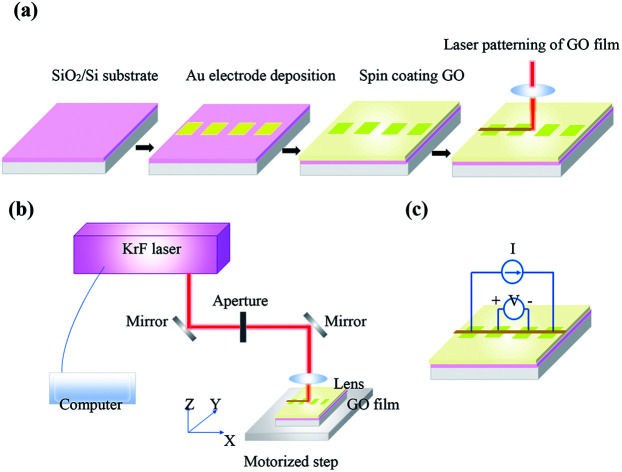

## 1. Introduction

Graphene has been the subject of much research because of its unique electrical, thermal and mechanical properties. It is a promising material in a wide variety of fields including optics and electronics, solar cells, light emitting devices, touch screens, flat panel displays and photovoltaic applications. Following exciting research on graphene, graphene oxide (GO) and reduced graphene oxide (rGO) have attracted great interest as a replacement of graphene in some aspects, like facile synthesis and potential applications in electronics and optoelectronics, circuits, sensors and supercapacitors.^1–24^ The Hummers method is the most promising fabrication process in which the precursor commonly undergoes a reduction process for mass production of the graphene-based material. RGO shows completely different chemical and physical properties compared to GO. Although a complete conversion of GO to graphene has proven to be difficult, partial rGO with improved electrical conductivity can be relatively easily obtained.^13–29^

In addition to the reduction process, many applications of the material require simultaneous patterning on various substrates. However, the common patterning methods need photoresist and fabrication of various masks to produce the desired patterns. A laser reduction method has been used to produce rGO patterns on GO films, which eliminates other complex methods such as lithography after primarily reduction process.^30,31^

So far, many studies have investigated laser patterning of graphene,^32–39^ graphite oxide^39,40^ and GO films.^41–46^ In particular, patterning GO films were performed using different lasers irradiation on various substrates.^41–49^ In one study,^29^ direct writing of conductive microcircuits on GO films was demonstrated using femtosecond laser irradiation. The removal of oxygen functional groups was confirmed by XPS studies and a reduction of electrical resistance was observed in rGO films. However, there isn't any evidence of transformation to the graphene-like structures in irradiated GO films, because no increase of 2D band was observed in Raman spectrum of irradiated GO film. The increase of the band intensity confirms transformation to the graphene-like structures. Another study^47^ of laser irradiation of GO films on a glass substrate was reported using a femtosecond laser with 800 nm wavelength. Nevertheless, the measured range of Raman spectra was limited just to D and G bands and there was no report of the 2D band at Raman spectra of their irradiated film. Several other authors have also looked at the interaction of KrF laser and GO film^48,50,51^ due to a close coincidence of the laser wavelength with those of chemical bonds of the material. In one study,^38^ producing the transparent circuit of reduced electrical resistance was reported on GO thin films on glass substrate. Nevertheless, any chemical analysis or structural investigations of irradiated GO film were not represented in this study. In another report^50^ the effect of KrF laser irradiation on GO films has been studied with a different number of laser pulses and laser fluences. The experiments were carried out in a vacuum and in a H_2_ atmosphere and the reduction process of GO films was found to be more efficient under H_2_ atmosphere than the vacuum. In a more recent report,^51^ laser reduction of GO films was studied using KrF, Ti:sapphire and CW lasers irradiation. The substrate used in their work was chosen to be polyethylene terephthalate (PET) because of its flexibility and suitability for supercapacitors. A comparison of the performance of each laser on a reduction of GO films at one certain laser fluence was reported and then, the effect of pulse duration on how reduction process of GO films was investigated. Raman and XPS data of GO films irradiated with KrF laser showed better results than those irradiated with femtoseconds and CW lasers. However, investigation of electrical resistance changes of irradiated GO films is absent in their study. The laser irradiation of GO films can result in fabrication of conductive circuits on insulator GO films that are attractive in microelectronic applications.

In the present study, the effect of KrF laser irradiation is investigated to improve various properties of GO films under different laser fluences in ambient atmosphere. SiO_2_/Si was chosen as a substrate because it is particularly attractive for microelectronics applications. A wide variety of characterization techniques was employed to monitor various properties of irradiated GO films included Raman spectroscopy, XPS, XRD, and AFM. These characterizations are necessary to completely understand the laser reduction process. More importantly, our study contains the investigation of the electrical resistance change of irradiated GO films under different laser fluence. The resultant rGO patterns of significantly different properties were obtained compared to the pristine GO films. We believe that our report is a comprehensive study of the laser irradiation process of GO thin films that includes characterization of various properties of irradiated films under similar irradiation conditions and on the same samples.

## 2. Experimental details

**Fig. 1:**
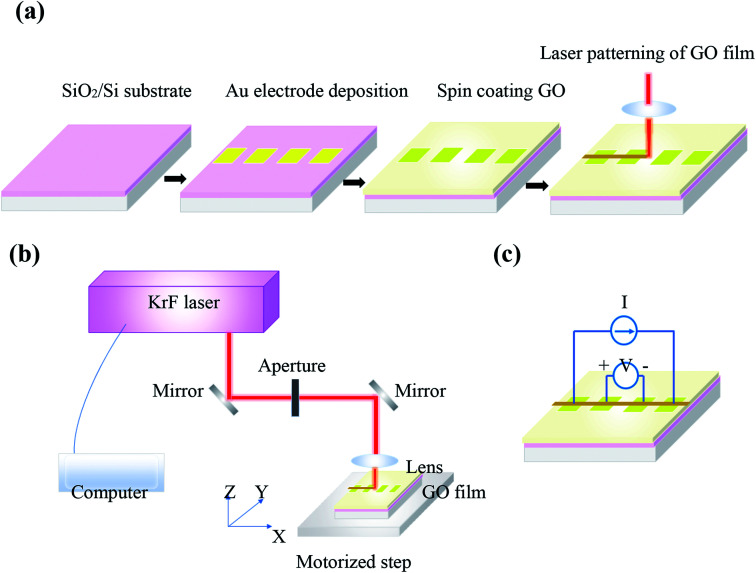
Schematic of (a) steps proposed to prepare GO films on SiO_2_/Si substrate, (b) KrF laser treatment system of the films and (c) electrical resistance measurements of irradiated films.

GO (Graphenea) used in the experiments was prepared *via* a modified Hummers method with a concentration 4 mg ml^−1^ in water. Gold electrodes were deposited on a SiO_2_/Si substrate for electrical resistance measurements. GO thin films were obtained by spin coating GO aqueous suspension on the substrate at 1000 rpm for 60 s and then dried at 60 °C. A pulsed KrF excimer laser of 248 nm wavelength and 10 ns pulse duration was used for the patterning of GO films. A schematic of steps proposed to prepare GO films and laser processing system is shown in Fig. 1(a and b), respectively. A 75 mm lens focuses the laser beam on the sample. GO film has been translated perpendicular to the laser beam and 100 μm s^−1^ scanning speed was chosen for laser treatment of samples. Laser treatment process of GO samples was carried out at ambient and at room temperature.

Raman spectrometer (Thermo Fisher, DXR) with a laser source 532 nm and a spot size 0.7 μm was used to characterize GO films. Surface topography was measured using atomic force microscope (AFM), model: Anasys NanoIR2, in the contact mode. X-ray diffraction (XRD) patterns were recorded using a Bruker X-ray diffractometer with Cu Kα irradiations operated at 40 kV and 30 mA. X-ray photoelectron spectroscopy (XPS) was employed to determine chemical states of GO films using a Physical Electronics PHI 5600 spectrometer with an Al Kα radiation source (1486.7 eV) with an energy resolution of FWHM 0.7 eV for sputtered clean Ag foil (Ag3d_5/2_) at pass energy 11.75 eV. De-convolution of all XPS data were performed using CasaXPS software with general forms of Gaussian and Lorentzian line shapes. Electrical resistance measurements were conducted using a 4-point probe method (Fig. 1(c)).

## 3. Results and discussion

**Fig. 2:**
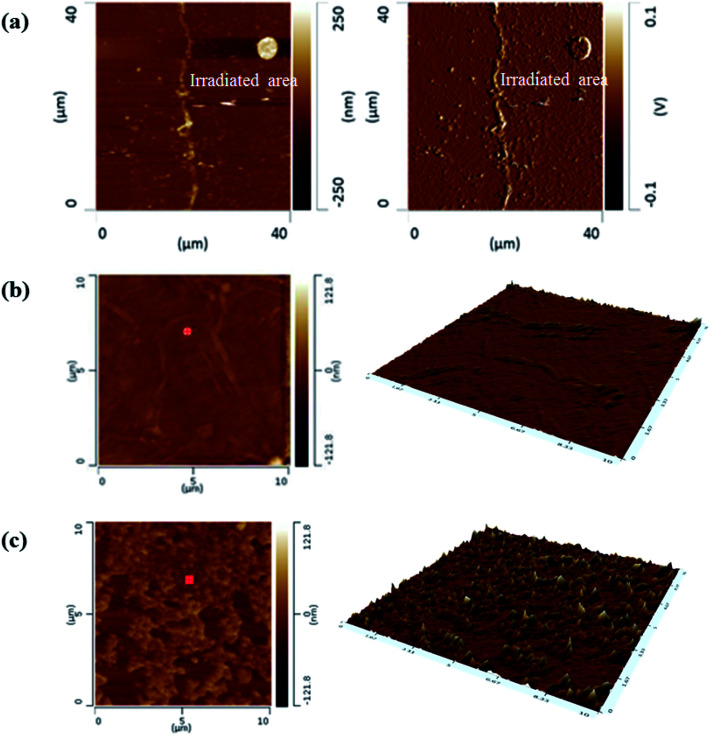
(a) AFM topography of GO film containing unirradiated and irradiated areas and (b and c) AFM 2D and 3D topography of unirradiated and irradiated areas in a 10 × 10 μm^2^ area scan, respectively.

Prepared GO films were exposed to KrF laser in an air atmosphere at laser fluences ranging from 4 to 72 mJ cm^−2^. Fig. 2(a) indicates AFM images of two separate areas of GO and irradiated GO at a certain laser fluence. The irradiation trace can be clearly observed in Fig. 2(a). Some islands also exist near irradiation trace due to slightly ablated GO layers that covered unirradiated area. AFM 2D and 3D topography of unirradiated and irradiated areas in smaller scan areas were shown in Fig. 2(b) and (c), respectively. AFM images show that the laser irradiation process increases roughness on the surface of irradiated GO film compared to the unirradiated area which showed a relatively smooth surface.

**Fig. 3:**
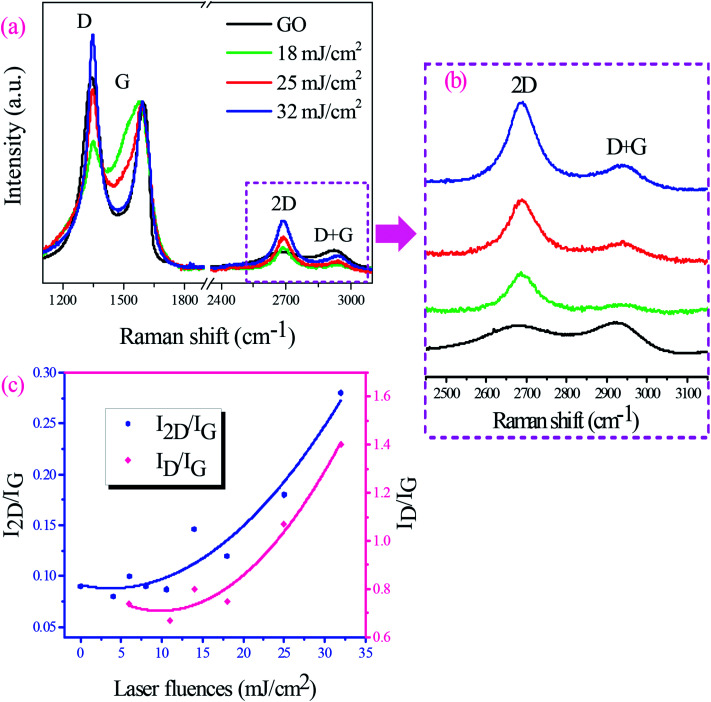
(a) Raman spectra of GO and rGO at several different laser fluences. All Raman spectra were normalized to G band, (b) the enlarged 2D region of rGO films at several different laser fluences and (c) dependence of *I*_2D_/*I*_G_ and *I*_D_/*I*_G_ ratios of GO and rGO films on laser fluence.

Raman spectroscopy was used to study laser reduction process of GO films. The typical Raman spectrum of GO shows the D and G bands around 1350 and 1580 cm^−1^. The D band is assigned to the out-of-plane breathing mode of the sp^2^ carbon atoms due to defects. The G band of graphene is produced by the in-plane vibration of C atoms and identifies the first order Raman-allowed mode of graphene.^25,47^ 2D band (2690 cm^−1^) is the second order of D band, however, there is no need for the presence of the defects for its activation. Fig. 3(a) compares Raman spectrum of GO film with those of GO films irradiated with laser fluences from 18 to 32 mJ cm^−2^. All Raman spectra were normalized to G band to more correctly evaluate changes of the 2D band with laser fluence: since this peak is known as a fingerprint of single-layer graphene. The Raman spectrum of the pristine GO film revealed a strong D band with the intensity comparable to the G band and a broad low-intensity 2D band. However, stronger and narrower 2D bands appeared in Raman spectra of irradiated GO films (Fig. 3(b)). The intensity of the peak increases as laser fluence goes up confirming the transformation to the graphene-like structures as a result of the laser irradiation process. It is established that *I*_D_/*I*_G_ ratio shows the degree of defects and *I*_2D_/*I*_G_ ratio indicates sp^2^ C=C bond in graphene structure. In order to a comparison of rGO films quality produced under various laser fluences, the trend of *I*_2D_/*I*_G_ and *I*_D_/*I*_G_ changes with increasing laser fluence was shown in Fig. 3(c). It is clear that increasing laser fluence causes a higher *I*_2D_/*I*_G_ ratio and it was expected that increasing laser fluence further results in higher values of *I*_2D_/*I*_G_. The increasing *I*_D_/*I*_G_ ratio in the figure also shows producing further defects during the laser irradiation process. Table 1 also summarizes the positions of each band, bands intensity ratios, and FWHM of the 2D band for rGO films at different laser fluences. The 2D band of irradiated GO films was centered at around 2690 cm^−1^. A decrease of FWHM of the 2D band was observed by increasing laser fluence as *I*_2D_/*I*_G_ ratio increased for GO film irradiated at these laser fluences. The least FWHM value 96.36 cm^−1^ and the highest *I*_2D_/*I*_G_ ratio 0.28 were calculated for GO film irradiated at 32 mJ cm^−2^ laser fluence. The lower value of *I*_D_/*I*_G_ ratio shows lesser defects and higher value of *I*_2D_/*I*_G_ ratio indicates the transformation to the graphene-like structures that results in higher charge mobility.

**Table 1:** Characteristics of Raman bands of GO and rGO films at different laser fluences

Laser fluence (mJ cm^−2^)	Band position (cm^−1^)			FWHM (cm^−1^)	Band intensity ratios		
	D	G	2D	2D	*I*_D_/*I*_G_	*I*_2D_/*I*_G_	*I*_2D_/*I*_D_
GO	1341	1597	2687	313.83	1.1	0.09	0.08
6	1345	1584	2687	305.79	0.74	0.1	0.13
14	1348	1584	2689	302.16	0.8	0.14	0.17
18	1349	1582	2686	239.28	0.75	0.12	0.16
25	1349	1591	2689	93.43	1.1	0.18	0.16
32	1347	1589	2690	96.93	1.4	0.28	0.2

**Fig. 4:**
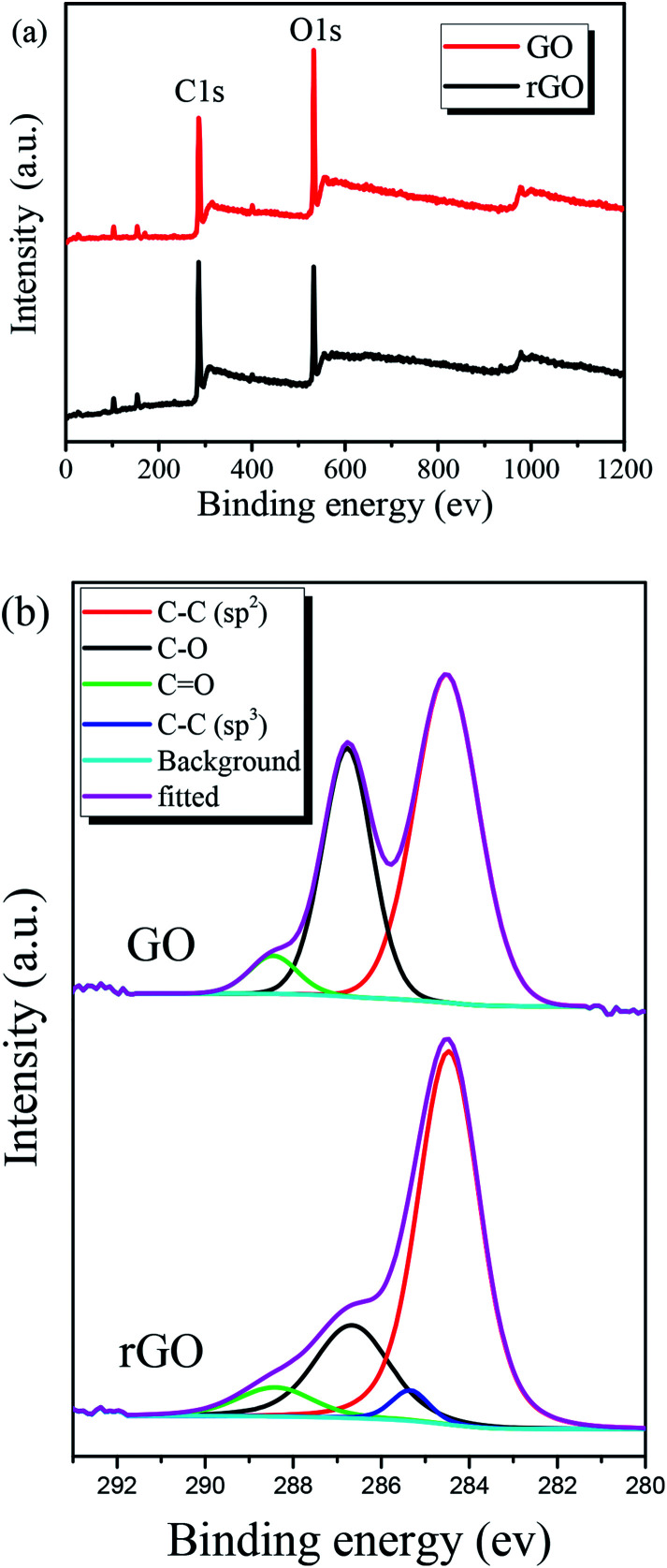
(a) XPS survey and (b) C1s XPS spectra of GO and rGO films at 32 mJ cm^−2^ laser fluence.

The chemical states of pristine GO film and GO film irradiated at 32 mJ cm^−2^ laser fluence were investigated by the use of XPS. Fig. 4(a) shows XPS results of the films and covers the carbon and oxygen regions. While pristine GO film represents C/O ratio of 0.65, the value reaches to about 1 after laser irradiation. The deconvolution of the C1s peak of the films is shown in Fig. 4(b). The C1s spectrum composed of reduced carbon species (∼284.5 and 285.6 eV), single bond carbon–oxygen components (∼286.7 eV) and double bond carbon–oxygen components (∼288.3 eV) as labeled in Fig. 4(b). In the XPS data of irradiated GO film, a strong peak corresponding to sp^2^ carbon bonds at 284.5 eV along with a small peak associated with the oxygenated functional groups are observed indicating the significant removal of oxygen contents. In addition, another lower intensity peak assigned to sp^3^ carbon bonds also exists in rGO film, which was also observed by others researchers.^50^

Table 2 represents C/O ratios and the atomic percentage of carbon components respectively derived from the XPS survey and C1s spectra for GO and rGO films. Based on the results, it seems KrF laser irradiation process mostly eliminates single bond carbon–oxygen components while the sp^2^ carbon bonds remain intact and then results in producing rGO patterns with a high C/O ratio.

**Table 2:** C/O ratios and atomic percentages of carbon components of GO and rGO films derived from XPS survey and C1s spectrum

	C/O	C–C (sp^2^ and sp^3^) (284.5 and 285.6 eV)	C–O (286.7 eV)	C=O (288.3 eV)
GO	0.65	43	37	20
rGO (32 mJ cm^−2^)	1.03	60	22	18

**Fig. 5:**
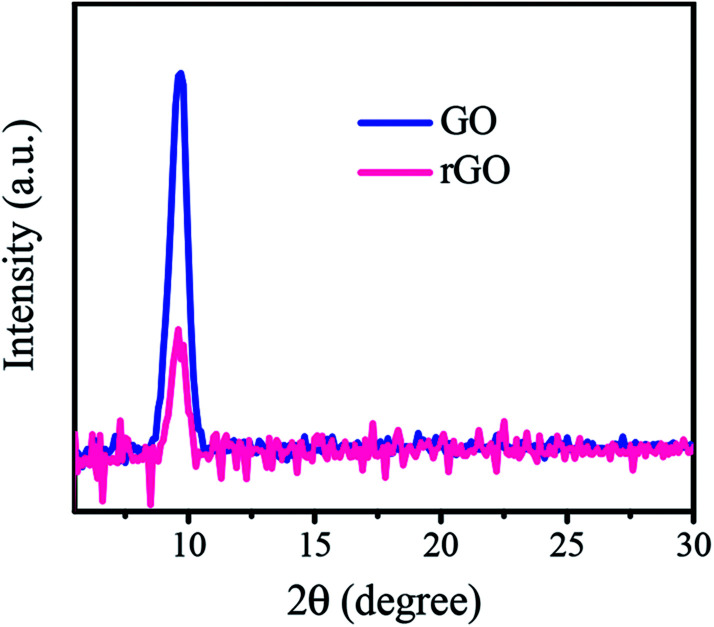
XRD patterns of GO and rGO films irradiated at 32 mJ cm^−2^ laser fluence normalized by Si peak.

To more carefully evaluate the reduction process of GO film on SiO_2_/Si substrate under laser irradiation, XRD patterns were recorded in a range of 2*θ* from 5° to 75°. The XRD pattern of graphite exhibits a peak located at ∼26.4°. This peak corresponds to a (002) reflection of graphite with a thickness of ∼3.37 Å of atomically flat graphene sheets. The position of the diffraction peak is shifted to a lower angle for GO due to increased interlayer spacing arising from the presence of oxygen functional groups. Fig. 5 compares the XRD pattern of rGO film at 32 mJ cm^−2^ laser fluence with that of GO film (only selected 2*θ* range shown in the figure). The XRD patterns show a diffraction peak at 2*θ* = 9.7° which refers to an interlayer spacing of 9.1 Å. Another peak also appears at 2*θ* = 69.3° related to Si substrate and was used to normalize the pattern. The XRD patterns of the GO and rGO films normalized by the Si peak. As observed, the sharp diffraction signal of GO was significantly reduced in intensity by laser irradiation which indicates removal of the oxygenated functional groups. On the other hand, there is no trace of graphite in GO and rGO films corresponding to the 2*θ* peak of 26.4°. Therefore, XRD observations are in agreement with the Raman and XPS results, confirming the transfer of GO to rGO. Similar results were obtained in other studies^31,50^ where they used glass and quartz as the substrate.

**Fig. 6:**
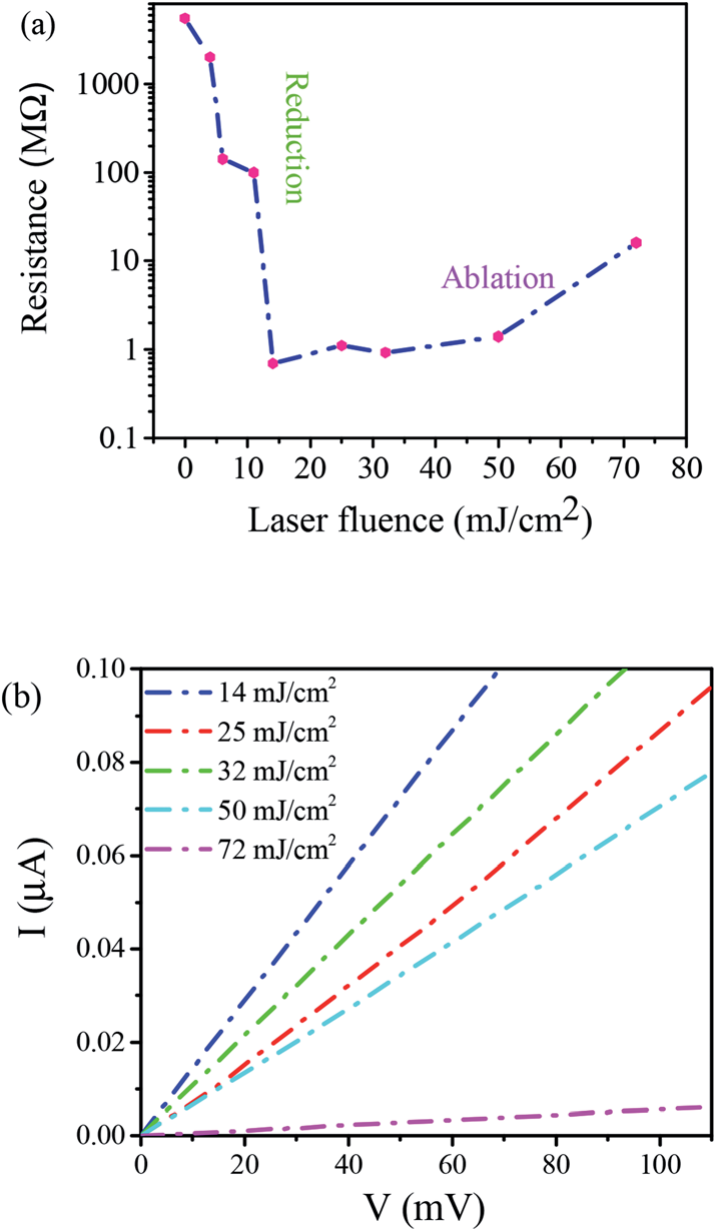
(a) Electrical resistance of rGO films as a function of laser fluence and (b) *I*–*V* curves of microcircuits produced at different laser fluences.

Further evidence of confirming of GO films reduction by laser irradiation was obtained using monitoring their electrical properties. The electrical behavior of the rGO films was studied by measuring the electrical resistance of the films irradiated at various laser fluences. Electrical resistance measurements of rGO films were conducted using a 4-point probe method. However, the electrical resistance of pristine GO films was measured by common method since they had a primarily high resistance of the order of GΩ. Fig. 6(a) shows the dependence of electrical resistance of the thin films on laser fluences. As observed, laser irradiation at low laser fluences caused a sharp drop of the electrical resistance of GO films. It was labeled as a reduction region in the figure. Since the electrical resistances of GO films strongly depend on the oxygen content, this can be explained by a comparison of 248 nm photons energy (5 eV) of the KrF laser with those of C and O bonds in GO. Since C=C and C=O bonds (6.36 and 7.69 eV, respectively) are much stronger than C–C and C–O bonds (3.61 and 3.73 eV, respectively), the energy of the photons of the KrF laser causes breaking C–C and C–O bonds and so electrical resistance significantly drops. A reduction of resistance by four orders of magnitudes was obtained at laser fluence from 14 to 32 mJ cm^−2^. However, the higher values of laser fluence cause a slight increase in resistance (ablation region). Therefore, laser irradiation at lower laser fluences results in reducing GO films, whereas at slightly higher values GO film is ablated to some extent which, in turn, causes the increase of electrical resistance, as were explained by Yung *et al.*^48^

The results of measured *I*–*V* characteristics to determine electrical resistances of rGO films at different laser fluences were presented in Fig. 6(b). Linear dependence between current and voltage shows stable conductivity of microcircuits produced under different laser fluences. These results show the capability to control the electric properties of GO films through an insulator to conductor transition by changing laser fluence.

## 4. Conclusions

In summary, laser irradiation of GO films was studied under various laser fluences in the air environment. Raman spectra of irradiated GO films showed the sharper and narrower 2D band with increasing laser fluence confirming transformation to the graphene-like structures upon laser processing. Moreover, laser irradiation of GO films resulted in the considerable elimination of the oxygenated functional groups as confirmed by XPS of irradiated GO films. Finally, KrF laser irradiation process of GO films resulted in fabrication of conductive rGO microcircuits with the reduced electrical resistances that can be used in microelectronics devices.

## Conflicts of interest

There are no conflicts to declare.

## Supplementary Material
